# Epigallocatechin-3-Gallate Ameliorates Acute Lung Damage by Inhibiting Quorum-Sensing-Related Virulence Factors of *Pseudomonas aeruginosa*

**DOI:** 10.3389/fmicb.2022.874354

**Published:** 2022-04-25

**Authors:** Huaqiao Tang, Suqi Hao, Muhammad Faraz Khan, Ling Zhao, Fei Shi, Yinglun Li, Hongrui Guo, Yuanfeng Zou, Cheng Lv, Jie Luo, Ze Zeng, Qiang Wu, Gang Ye

**Affiliations:** ^1^Department of Pharmacy, College of Veterinary Medicine, Sichuan Agricultural University, Chengdu, China; ^2^Department of Botany, Faculty of Basic and Applied Sciences, University of Poonch Rawalakot, Rawalakot, Pakistan; ^3^National Ethnic Affairs Commission Key Open Laboratory of Traditional Chinese Veterinary Medicine, Tongren Polytechnic College, Tongren, China; ^4^Engineering Research Center of the Medicinal Diet Industry, Tongren Polytechnic College, Tongren, China; ^5^Agricultural College, Yibin Vocational and Technical College, Yibin, China

**Keywords:** anti-virulence, quorum sensing, EGCG, *P. aeruginosa*, acute lung infection

## Abstract

The superbug *Pseudomonas aeruginosa* is among the most formidable antibiotic-resistant pathogens. With declining options for antibiotic-resistant infections, new medicines are of utmost importance to combat with *P. aeruginosa*. In our previous study, we demonstrated that Epigallocatechin-3-gallate (EGCG) can inhibit the production of quorum sensing (QS)-regulated virulence factors *in vitro*. Accordingly, the protective effect and molecular mechanisms of EGCG against *P. aeruginosa*-induced pneumonia were studied in a mouse model. The results indicated that EGCG significantly lessened histopathological changes and increased the survival rates of mice infected with *P. aeruginosa*. EGCG effectively alleviated lung injury by reducing the expression of virulence factors and bacterial burden. In addition, EGCG downregulated the production of pro-inflammatory cytokines, such as TNF-α, IL-1, IL-6, and IL-17, and increased the expression of anti-inflammatory cytokines IL-4 and IL-10. Thus, the experimental results supported for the first time that EGCG improved lung damage in *P. aeruginosa* infection by inhibiting the production of QS-related virulence factors *in vivo*.

## Introduction

Globally, pneumonia is a severe public health problem with a large disease burden and a major cause of mortality and morbidity. The disease burden caused by pneumonia affects more children worldwide than other diseases (O’Brien et al., 2019). Older patients have a high risk of death afflicted by pneumonia (Quinton and Mizgerd, 2015). Some of the bacterial and viral infections causing acute pneumonia or sepsis result in serious inflammatory damage to the lungs, thereby leading to the progression of acute lung injury (ALI) or acute respiratory distress syndrome (ARDS), especially in critically ill patients (Dai et al., 2018). Multidrug-resistant microbes (“superbugs”), such as *Streptococcus pneumoniae*, group A *Streptococcus, Klebsiella* pneumoniae, *Staphylococcus aureus*, *Mycoplasma pneumoniae*, and *Pseudomonas aeruginosa* can cause severe pneumonia and were the main inducers of acute pathogenic factors (Ravi Kumar et al., 2018).

*P. aeruginosa*, a common opportunistic pathogen, can cause life-threatening respiratory infections and is responsible for hospital-acquired infections and ventilator-associated pneumonia (VAP). The high adaptability and an increasing number of multidrug-resistant *P. aeruginosa* constitute a threat for those who suffer from Chronic Obstructive Pulmonary Disease (COPD) or Cystic Fibrosis (*CF*; Aloush et al., 2006). Unfortunately, because of its intrinsic and extrinsic drug resistance and the capacity of *P. aeruginosa* to form biofilms, a *P. aeruginosa* infection is notoriously difficult to treat with antibiotics (Moore and Flaws, 2011). Multidrug-resistant pathogen-induced death is estimated to reach 10 million by 2050, and thereby exceeding deaths caused by cancer and diabetes combined worldwide. Thus, the development of alternative therapeutic methods is critical (O’Neill, 2016). The QS system is a cell-to-cell system that enables microbe populations to change their behavior based on population density. The QS system of *P. aeruginosa* plays a key role in coordinating various activities, including biofilm formation and the release of virulence factors. The QS system of *P. aeruginosa* is mainly regulated by four QS network subsystems, including *lasI/lasR, rhlI/rhlR*, PQS, and IQS systems (Lee and Zhang, 2015).

*P. aeruginosa*-induced pneumonia expresses a myriad of virulence factors, including flagella, pili, lipopolysaccharides (LPS), elastase, alkaline phosphatase, exotoxin A, as well as components of the type III secretion system (T3SS; Sadikot et al., 2005). In addition, the ability of *P. aeruginosa* to form biofilms is conducive to establishing infections in VAP and *CF* patients and is difficult to eradicate (Costerton et al., 1999). *P. aeruginosa*-induced pneumoniae is a complex process. Several surface-associated elements, including flagella, fimbriae, and LPS, contact host respiratory epithelia through a number of aggregated pilis (Gellatly and Hancock, 2013). Once contact with host epithelia has occurred, T3SS can be activated. *P. aeruginosa* T3SS is a major determinant of virulence, and its expression is frequently associated with acute invasive infections. Moreover, *P. aeruginosa* T3SS has been linked to increased mortality in infected patients (Hauser, 2009). Elastase (*lasB lasA*) has been shown to directly injure lung tissue through disruption of epithelial tight junctions and basal membranes. Elastase may increase the recruitment of neutrophils into the airways, which can result in serious inflammation (Kipnis et al., 2006). Pyocyanin induces direct damage to the respiratory tract as epithelial necrosis, and slowing tracheal mucociliary transport results from damaged ciliary movement (Lau et al., 2004). Virulence factors, such as type III secretory proteins, QS systems, and LPS, activate the host immune response. Therefore, the activation of macrophages, neutrophilic granulocytes, and T cells induces the secretion of cytokines, chemotactic factors, and another inflammatory mediator, thus leading to lung injury and mortality (Driscoll et al., 2007).

For thousands of years, tea originating from China has gained the world’s taste. It has become the daily health drink for many individuals. In general, these health benefits result from the phenolic compounds that are present in green tea, particularly catechins. Numerous studies have demonstrated the diverse activities of Epigallocatechin-3-gallate (EGCG), including antioxidant, antibacterial, antiviral, antitumor and anti-inflammatory activities. In our previous study, we demonstrated that EGCG significantly acted against the expression of *P. aeruginosa* QS-regulated virulence *in vitro*, and significantly increased the survival rate of *Caenorhabditis elegans* infected with *P. aeruginosa* (Hao et al., 2021). QS quenching, called anti-virulence therapy, has been considered a suitable strategy to settle the multidrug resistance problem (Clatworthy et al., 2007). Taken together, our study provides additional support for drinking tea as an effective method against bacterial infections. These findings can pave the way for the use of EGCG as a therapeutic medicine to treat bacterial infections in the lungs.

## Materials and Methods

### Animals, Bacterial Strains, and Chemicals

Eight-week-old male ICR mice (25–30 g) were purchased from Sibeifu Biotechnology Co. Ltd. (Beijing, China). Mice were housed at 22–25°C with a 12 h day-night cycle, were fed standard rodent chow, and sterile water *ad libitum* for 1 week of acclimation. All protocols involving animal studies were reviewed and approved by the Animal Ethical Committee of Sichuan Agricultural University (#20210020). *P. aeruginosa* (PAO1) were cultured in Luria Bertani (LB) medium (Sangon Biotech, Shanghai, China). EGCG was obtained from Shanghai Yuanye Bio-Technology Co., Ltd. (Shanghai, China); CIP was obtained from Sichuan Chuanlong Dongke Pharmaceutical Co., Ltd.

### Experimental Protocol

A total of 90 mice were randomly divided into six groups (*n* = 15 mice per group): a control group, PAO 1 group, PAO 1 + EGCG (20 mg/kg) group, PAO 1 + EGCG (40 mg/kg) group, PAO 1 + EGCG (80 mg/kg) group, and a Ciprofloxacin (CIP; 20 mg/kg) group. CIP was used as positive control. Mice were administered the same volume of saline or drugs by intragastric administration for 3 days. Mice were infected by using a previously described method with modifications (Traber et al., 2019). Briefly, mice were weighed and anesthetized, and received intratracheal instillation of PAO 1 (2.5 × 10^8^ CFU) in 20 μl phosphate buffered saline (PBS) to model an acute infection with PAO 1. Mice were anesthetized and sacrificed at 24 h after infection. Samples were harvested rapidly for subsequent analysis. Per group, 10 mice were randomly selected to collect blood and lung tissue; another five mice were selected for the collection of bronchial-alveolar lavage fluid (BALF).

### Colony Counting of the Lungs

To assess bacterial burden in the lung, fresh lung tissues were extracted aseptically, and homogenized in sterile NaCl 0.9%. As previously described (Pylaeva et al., 2019), colony forming units (CFU) were enumerated by plating serial dilutions of lung homogenates on *Pseudomonas* Agar Medium for the detection of Pyocyanin (PDP, Haibo Biotech, Shandong, China). After incubation at 37°C for 24 h, CFU were counted and recorded.

### Histopathological Evaluation

Lung tissue was fixed with 4% paraformaldehyde overnight, and embedded in paraffin. Subsequently, lung tissue was cut into 5-μm-thick sections. Sections were stained with hematoxylin and eosin (H&E) and the degree of damage to lung tissue was validated (Sen-Kilic et al., 2019).

### ELISA

BALF was collected and centrifuged at 1500 g for 5 min, then the supernatant was removed and transferred to a clean tube. The expression of TNF-α, IL-10, and IL-17 in the supernatant was determined by ELISA according to the manufacturer’s instructions [Multisciences (Lianke) Biotech Co., Ltd., Hangzhou, China]. The absorbance was measured using a microplate reader at 450 nm.

### RNA Extraction and qPCR

Total RNA was extracted from lung tissue homogenate using TRIzol reagent (Songon Biotech, Shanghai, China) according to the manufacturer’s instructions. Then, cDNA synthesis was conducted using a RevertAid First Strand cDNA Synthesis kit (Thermo Fisher Scientific, K1622, Waltham, MA, United States) in a 20 μl volume of reaction mixture. Real-time PCR was performed in a total reaction volume of 10 μl, containing 5 μl PerfectStartTM Green qPCR SuperMix (TransGen Biotech, Beijing, China), 2 μl template cDNA, 1 μl of primers (Shenzhen Huada Gene Research Institute, Shenzhen, China), and 2 μl DNase/RNase-free water (Tiangen Biotech, Beijing, China). Finally, pvdQ of *P. aeruginosa* was chosen as the reference gene. The qRT-PCR reaction was conducted on a LightCycler^®^ 480II Master Mix (Roche, Germany). Data from qRT-PCR experiments were analyzed using the 2^®-ΔΔCT^ method (Yang et al., 2021). Primer sequences are listed in Table 1.

**Table 1:** PCR primers of virulence factors and inflammatory cytokines.

Primer name	Type	Primer sequence
lasI	Fw	CGCACATCTGGGAACTCA
	Rev	CGGCACGGATCATCATCT
lasR	Fw	CTGTGGATGCTCAAGGACTAC
	Rev	AACTGGTCTTGCCGATGG
rhlI	Fw	GTAGCGGGTTTGCGGATG
	Rev	CGGCATCAGGTCTTCATCG
rhlR	Fw	GCCAGCGTCTTGTTCGG
	Rev	CGGTCTGCCTGAGCCATC
pqsA	Fw	GACCGGCTGTATTCGATTC
	Rev	GCTGAACCAGGGAAAGAAC
pqsR	Fw	CTGATCTGCCGGTAATTGG
	Rev	ATCGACGAGGAACTGAAGA
phzM	Fw	ACGGCTGTGGCGGTTTA
	Rev	CCGTGACCGTCGCATT
lasA	Fw	CTGTGGATGCTCAAGGACTAC
	Rev	AACTGGTCTTGCCGATGG
lasB	Fw	AACCGTGCGTTCTACCTGTT
	Rev	CGGTCCAGTAGTAGCGGTTG
phzA	Fw	AACGGTCAGCGGTACAGGGAAAC
	Rev	ACGAACAGGCTGTGCCGCTGTAAC
phzH	Fw	GCTCATCGACAATGCCGAACT
	Rev	GCGGATCTCGCCGAACATCAG
phzS	Fw	CCGAAGGCAAGTCGCTGGTGA
	Rev	GGTCCCAGTCGGCGAAGAACG
RhlA	Fw	TGGCCGAACATTTCAACGT
	Rev	GATTTCCACCTCGTCGTCCTT
RhlC	Fw	GCCATCCATCTCGACGGAC
	Rev	CGCAGGCTGTATTCGGTG
rpod	Fw	GGGCGAAGAAGGAAATGGTC
	Rev	CAGGTGGCGTAGGTGGAGAA
TNF-α	Fw	CTTCTGTCTACTGAACTTCGGG
	Rev	CAGGCTTGTCACTCGAATTTTG
IL-1	Fw	GAAATGCCACCTTTTGACAG
	Rev	TGGATGCTCTCATCAGGACAG
IL-6	Fw	TAGTCCTTCCTACCCCAATTTCC
	Rev	TTGGTCCTTAGCCACTCCTTC
IL-4	Fw	GGTCTCAACCCCCAGCTAGT
	Rev	GCCGATGATCTCTCTCAAGTGAT
IL-10	Fw	GCTGGACAACATACTGCTAACC
	Rev	ATTTCCGATAAGGCTTGGCAA
IL-17	Fw	TTTAACTCCCTTGGCGCAAAA
	Rev	CTTTCCCTCCGCATTGACAC
GAPDH	Fw	TCAACGGCACAGTCAAGGC
	Rev	CTCCACGACATACTCAGCACC

### Statistical Analysis

Data are expressed as the mean ± standard deviation (SD), and all experimental groups were compared with the control group. Data were analyzed using SPSS 20.0 by ANOVA (IBM SPSS Statistics, CA, United States), and *p* < 0.05 or *p* < 0.01 was considered significantly different.

## Results

### Protective Efficacy of EGCG Against Acute Lung Infection

**Figure 1:**
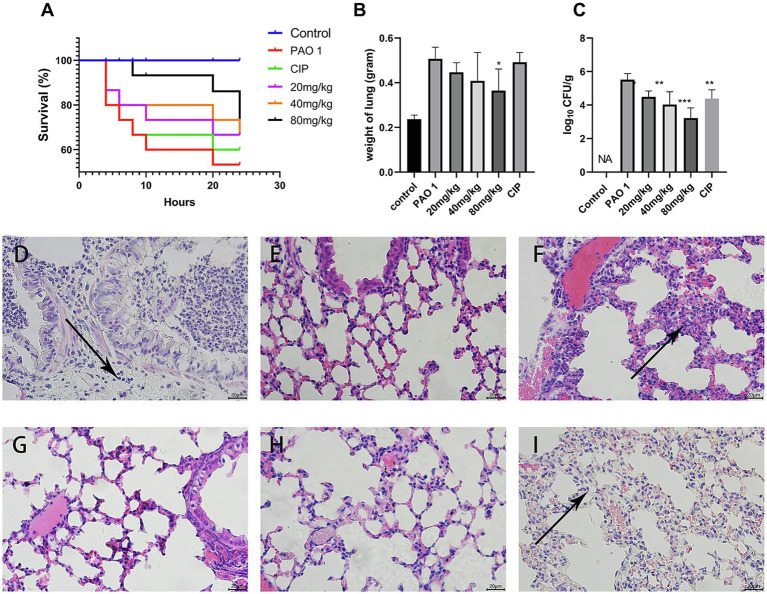
Protective efficacy of EGCG against acute lung infection. **(A)** The survival rate was measured at different time points post *P. aeruginosa* challenged. **(B)** The weight of the lungs after *P. aeruginosa* challenged. **(C)** Bacterial load in the lungs were evaluated at 24 h post infection. **(D–I)** Pathological changes in **(D)** *P. aeruginosa* group, **(E)** control group, **(F)** EGCG (20 mg/kg) group, **(G)** EGCG (40 mg/kg) group, **(H)** EGCG (80 mg/kg) group, **(I)** CIP was used as positive control. Data are presented as mean ± SD and analyzed with one-way ANOVA. ^*^p < 0.05, ^**^p < 0.01, ^***^p < 0.005 vs. PAO 1 group.

As shown in Figure 1, the survival of mice with a *P. aeruginosa*-induced acute lung infection was evaluated to test the protective effect of EGCG. Of the mice in the *P. aeruginosa* group, only 53.4% survived. The survival rates after EGCG early intervening treatment with 80 mg/kg, 40 mg/kg, 20 mg/kg, respectively, were 71.8, 66.7, 66.7%, whereas the survival rate of the positive CIP group was 60%, thus the protective effect was less compared to that of EGCG. Next, the alleviation of *P. aeruginosa*-induced pulmonary edema by EGCG was assessed. The results showed that the wet weight of mice in the *P. aeruginosa* group increased more than 2-fold compared with mice in the control group. EGCG significantly reduced the lung edema, and no significant differences in lung weight were observed between CIP and *P. aeruginosa*. The bacterial burden in the lungs was determined by plating serial dilutions and counting viable bacteria at 24 h post the challenge. As expected, the bacterial load in the lungs of EGCG-treated mice was significantly lower compared to that of mice in the *P. aeruginosa* group. These findings demonstrated the preventive effect of EGCG in decreasing acute lung infection of *P. aeruginosa*. The control group did not show any histopathological changes in the lungs under the light microscope. The results indicated that lung sections after *P. aeruginosa* infection showed significant changes, including PMN infiltration, alveolar interstitial edema, interstitial hemorrhage, and alveolar wall thickening. Importantly, our results showed that EGCG treatment reduced lung damage and maintained alveolar integrity due to structural disruptions and hemorrhage in a concentration-dependent manner compared with the control. Taken together, these results demonstrated that EGCG alleviated histopathological damage in lungs of mice challenged with *P. aeruginosa*.

### EGCG Alleviated the Expression of QS System-Regulated Genes *in vivo*

**Figure 2:**
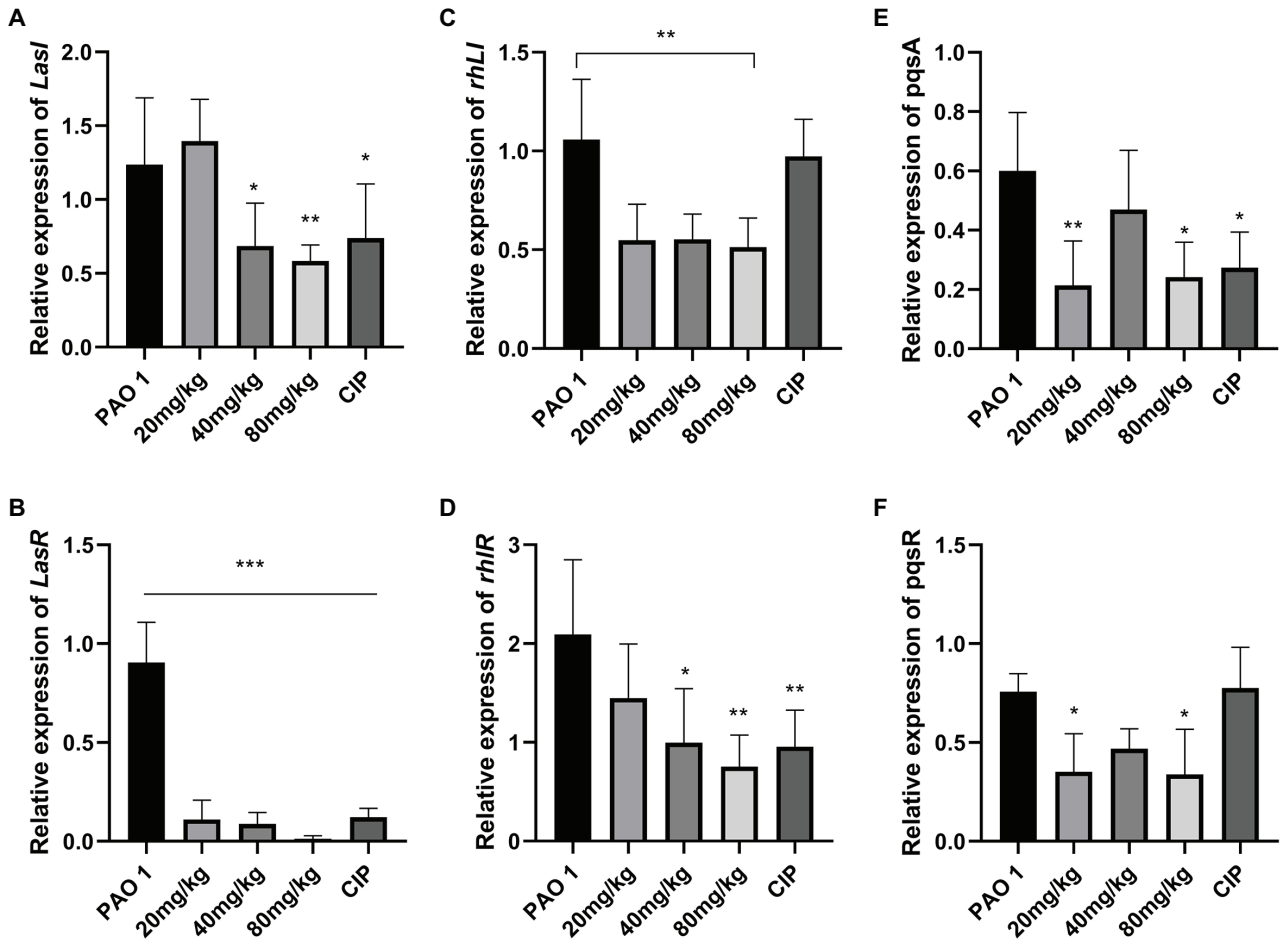
The effect of EGCG on the expression of QS system *in vivo*. **(A)** *lasI*, **(B)** *lasR*, **(C)** *rhlI*, **(D)** *rhlR*, **(E)** *pqsA*, and **(F)** *pqsR*. Data are presented as mean ± SD and analyzed with one-way ANOVA. ^*^p < 0.05, ^**^p < 0.01, ^***^p < 0.005 vs. PAO 1 group.

**Figure 3:**
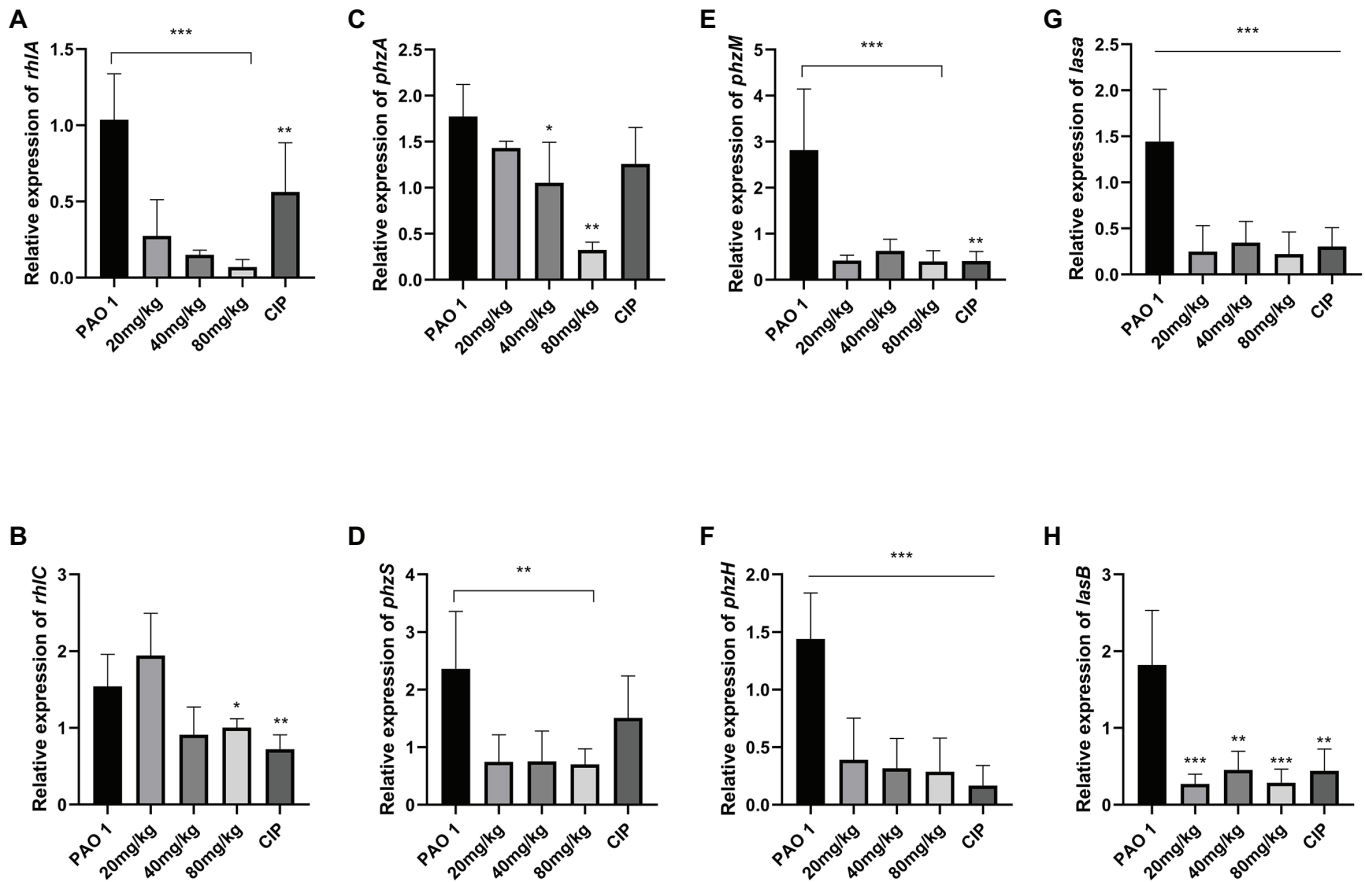
The inhibition of EGCG on the expression of QS-regulated virulence factors in the lung challenged by *P. aeruginosa*. **(A)** *rhlA*, **(B)** *rhlC* **(C)** *phzA*, **(D)** *phzS*, **(E)** *phzM* **(F)** *phzH*, **(G)** *lasA*, **(H)** *lasB*. Data are presented as mean ± SD and analyzed with one-way ANOVA. ^*^p < 0.05, ^**^p < 0.01, ^***^p < 0.005 vs. PAO 1 group.

In this study, the expression of QS system genes was examined in acute lung injury. As shown in Figure 2, EGCG and CIP significantly downregulated the expression of QS-related virulence factors *in vivo*. First, EGCG inhibited the master QS regulatory system *lasI/lasR* in a dose-dependent manner. The expression of *lasR* was significantly inhibited at 80 mg/kg. At a dose of 80 mg/kg, the expression of *lasA* and *lasB*, which are regulated *via* the *lasI*/*lasR* pathway, was also significantly inhibited in a dose-dependent manner. Moreover, *rhlI/rhlR* and *pqsA/pqsR* systems were downregulated as well. Our data showed that the expression level of the *pqsA/pqsR* system was lower than that of *lasI*/*lasR* and *rhlI/rhlR* systems. Therefore, we hypothesized that *lasI*/*lasR* and rhLI/rhlR systems played a dominant role in acute lung infection (Figure 3). The expression of virulence factors was significantly downregulated by EGCG. The expression of *phzA*, *phzS*, *phzM*, and *phzH* was also inhibited by EGCG compared with mice in the *P. aeruginosa* group. Furthermore, the *rhlA* gene, which controls the production of rhamnolipid, was significantly inhibited.

### EGCG Inhibited Biofilm Expression *in vivo*

**Figure 4:**
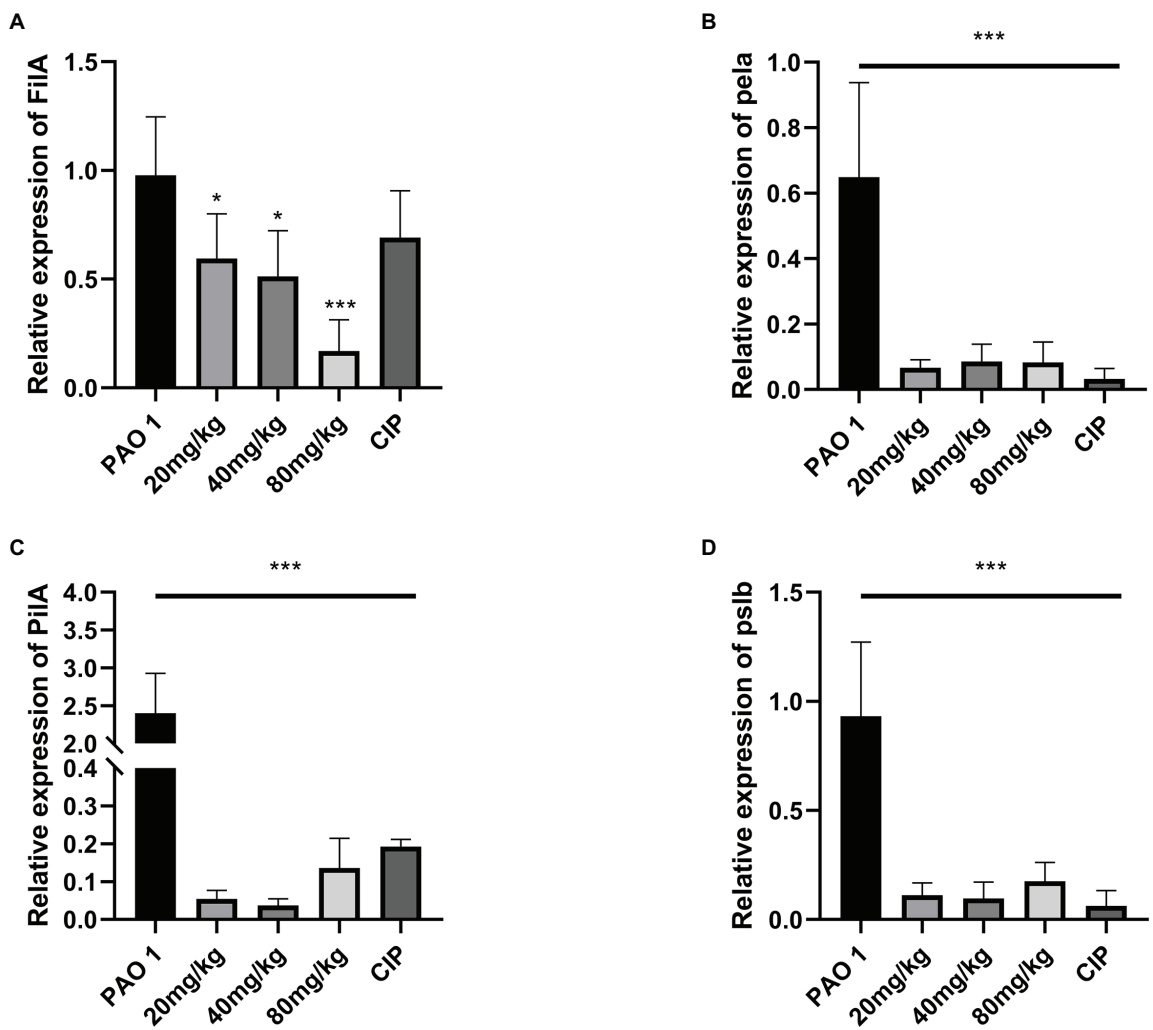
EGCG significantly affected the expression of the biofilm formation relevant genes *in vivo*. **(A)** *FilA*, **(B)** *pela* **(C)** *PilA*, **(D)** *pslb*. Data are presented as mean ± SD and analyzed with one-way ANOVA. ^*^p < 0.05, ^**^p < 0.01, ^***^p < 0.005 vs. PAO 1 group.

*Pseudomonas aeruginosa* biofilm formation is regulated by the QS system (Sauer et al., 2002). The expression of biofilm matrix genes and subsequent development of biofilm structure are also adjusted by the QS system (Skariyachan et al., 2018). The expression of biofilm-regulated factors (*fila, pela, pila, pslb*) was evaluated *in vivo*. The results indicated that EGCG significantly inhibited biofilm maturation at 18 h *in vitro*, and a better inhibitory effect was found *in vivo*. The expression of *pela*, *pila*, and *pslb* was significantly inhibited by EGCG in comparison to mice in the *P. aeruginosa* infection group. Thus, these results suggested that EGCG can effectively inhibit biofilm formation and prevent the activation of virulence factors of *P. aeruginosa in vivo*. The data are presented in Figure 4.

### EGCG Inhibited Inflammation in *Pseudomonas aeruginosa*-Induced Lung Injury

**Figure 5:**
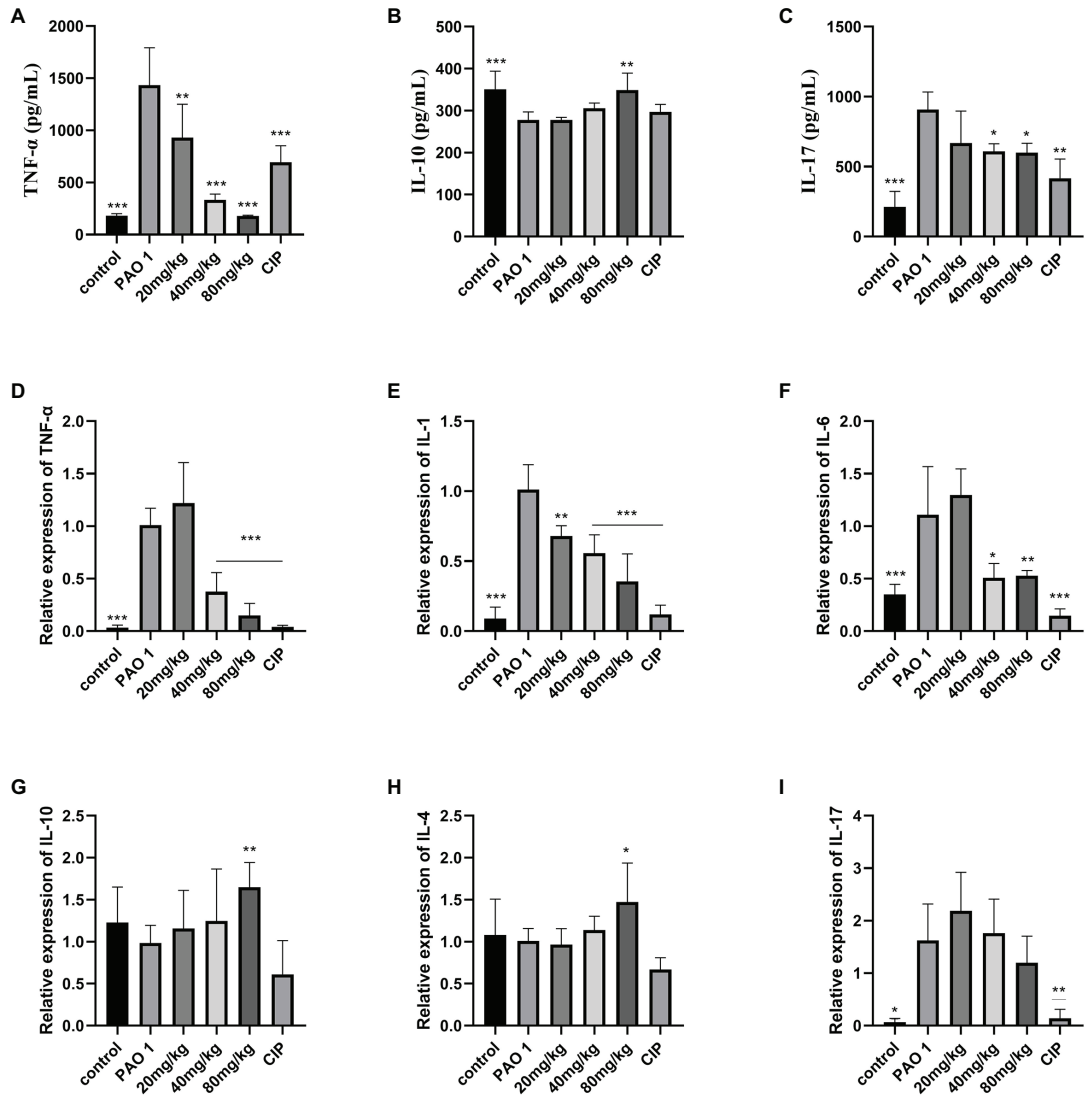
Assay of inflammatory cytokines by ELISA and QRT-PCR. Secreted TNF-α, IL-10, and IL-17 from the bronchial-alveolar lavage fluid was assessed by ELISA assay kit **(A–C)**. EGCG affected the expression of cytokines with a dosage dependent manner in the lung **(D–I)**. Data are presented as mean ± SD and analyzed with one-way ANOVA. ^*^p < 0.05, ^**^p < 0.01, ^***^p < 0.005 vs. PAO 1 group.

To assess the inflammatory state of infected mice, the expression of inflammatory cytokines was determined by real-time PCR and ELISA. Compared to the control, EGCG significantly decreased the expression of proinflammatory cytokines TNF-α, IL-1, IL-6, and IL-17. Moreover, the expression of proinflammatory cytokines in the CIP group was lower than that in the EGCG group. The EGCG group had a higher expression of anti-inflammatory cytokines IL-4 and IL-10 (Figure 5).

## Discussion

In this study, it was demonstrated that EGCG protects mice against *P. aeruginosa*-induced lung damage by inhibiting the virulence controlled by QS systems. *P. aeruginosa* is a major cause of acute nosocomial infections and pneumonia (Kizny Gordon et al., 2017). Nosocomial bacteremia pneumonia is associated with significant mortality, morbidity, length of hospital stays and cost. In addition, despite important medical advances, there have been no improvements in the treatment of *P. aeruginosa* infections over the past two decades (Melsen et al., 2013; Jain et al., 2015). The mechanisms by which *P. aeruginosa* resists antibiotics include intrinsic, acquired and adaptive resistance, such as efflux pumps, antibiotic-inactivating enzymes, impermeable outer membrane proteins, horizontal transfer of resistance genes or mutational changes, and adaptive resistance refers to the formation of biofilms (Livermore, 2002; Breidenstein et al., 2011). *P. aeruginosa* is resistant to diverse types of antibiotics, including quinolones, aminoglycosides, and β-lactams (Hancock and Speert, 2000). Due to the increasing frequency of antibiotic resistance of *P. aeruginosa*, novel therapeutic approaches to treat *P. aeruginosa* infections are of utmost importance. Furthermore, novel treatments may be used alone or in combination with conventional therapies, such as the inhibition of QS and bacterial lectins, the use of iron chelation, phage therapy, vaccine strategies, as well as the use of nanoparticles, antimicrobial peptides and electrochemical scaffolds (Chatterjee et al., 2016). In our previous study, the minimal inhibitory concentration (MIC) of EGCG against *P. aeruginosa* was confirmed to be 512 μg/ml (Hao et al., 2021). However, EGCG reduced virulence phenotypes, such as biofilm, protease, elastase activity, swimming, and swarming motility at concentrations where no growth inhibition was observed. In the present study, we aimed to explore the anti-infection ability of EGCG based on the QS mechanism in a mouse model of *P. aeruginosa* infection. Our results indicated that EGCG significantly alleviated *P. aeruginosa*-induced pulmonary edema, decreased the bacterial load and the level of proinflammatory cytokines in the lung, and enhanced the survival rates of mice. Moreover, EGCG significantly reduced the expression of QS-regulated virulence factors *in vivo*. Taken together, these findings showed that EGCG has the ability to mitigate the release of virulence and may alleviate inflammation-caused damage *in vivo*.

EGCG is one of the richest ingredients in green tea-derived polyphenols, and its biological activities have been extensively studied (Jigisha et al., 2012; Steinmann et al., 2013). EGCG can inhibit infections by reducing biofilm formation and toxin release in bacteria (Zhao et al., 2021). However, few studies have evaluated the clinical effect of EGCG in animal models. In several previous studies, the anti-inflammation effect of EGCG was demonstrated. Lee et al. demonstrated that EGCG protected against TNF-α-mediated lung inflammation by down-regulation of oxidative stress and expression of intercellular adhesion molecule (ICAM)-1 in A549 cells or human pulmonary alveolar epithelial cells (HPAEpiCs) as well as in mouse lungs (Lee et al., 2013). In a recent study, the effect of green tea (GTE) and EGCG on macrophage polarization was evaluated *in vitro* and it was determined whether the treatment could ameliorate inflammatory responses *in vivo*. Results showed that GTE and EGCG decreased M1-macrophages and increased Treg cells in bone marrow to inhibit inflammation. EGCG and GTE prevent LPS-induced inflammatory damage contributing to restore the immune system homeostasis through increasing M2-macrophages, N2-neutrophils and Tregs in the spleen and blood (Azambuja et al., 2022). The activation of M2 macrophages, which polarized by Th2 cytokines such as IL-4 and IL-13, can enhance the effect anti-inflammatory and immunoregulatory. Consequently, increasing the release of anti-inflammatory cytokines IL-10 and TGF-β (Shapouri-Moghaddam et al., 2018). Wang et al. (2019) demonstrated that pretreatment with EGCG attenuated LPS-induced ALI as manifested by fewer pathological changes in pulmonary edema and the expression of proinflammatory cytokines TNF-α, IL-1β, and IL-6 in the lung, serum, and BALF. The protective mechanism was associated with suppressing the TLR-4/NF-kB-p65 pathway that mediates inflammation.

These results were similar to the data obtained in our study; we demonstrated that EGCG alleviates lung damage, including pathological injury and pulmonary edema in *P. aeruginosa*-induced pulmonary infection. Moreover, EGCG decreased the *P. aeruginosa* load and infection mortality in the lung. EGCG significantly inhibited the expression of TNF-α, IL-1β, IL-6, and IL-17 in the lung but increased the expression of anti-inflammatory cytokines IL-4 and IL-10. We speculate that EGCG may improve the immunity ability through promoting the polarization of M2 macrophages. Hence EGCG increased the level of anti-inflammatory cytokines IL-4 and IL-10. Further work needs to be performed to identify the underlying mechanisms involved. The ELISA results showed that the protein expression levels of TNF-α and IL-17 decreased in a dose-dependent manner, compared with PAO 1 group. Therefore EGCG can reduce *P. aeruginosa*-induced inflammation in the lung. Liu et al. demonstrated that tea polyphenols increased the survival rate of Caenorhabditis elegans against K. pneumonia infection to 73.3 and 82.2% (Liu et al., 2020). Previous results showed that microencapsulation of EGCG exhibited therapeutic outcomes by resolution of inflammation in tuberculosis bacteria-infected lungs and a significant reduction in bacterial burden (Sharma et al., 2020). Many studies have suggested that EGCG and other polyphenol compounds could be potential antivirulence agents for pulmonary infection (Sriram et al., 2009; Ling et al., 2012). The potent anti-inflammatory activities add further appeal to the medicinal use of EGCG or other green tea polyphenol-rich products.

*P. aeruginosa* can express a plethora of virulence factors that facilitate invasion and damage host tissues (Crousilles et al., 2015), and are controlled by complex, intersecting regulatory circuits and multiple signaling systems (Nadal Jimenez et al., 2012; Balasubramanian et al., 2013). Among these, QS regulates virulence factors (proteases, elastase, rhamnolipid, exotoxins, and pyocyanin) and plays an important role in acute *P. aeruginosa* infections (Hauser, 2009; Lee and Zhang, 2015). QS participates in biofilm formation, which plays an important role during chronic infections and antibiotic resistance. In recent years, antimicrobial agents with great potential have been studied and are now known as antivirulence therapies (Hauser, 2009). This intervention targets virulence factors or virulence regulatory pathways that will not result in inhibition of bacterial growth or bacterial cell death but block their pathogenicity (Hauser, 2009). Consequently, the emergence of drug-resistant strains is decreasing. Interference with QS-mediated signaling and alleviation of virulence contribute to clearance of infecting bacteria by host defenses (Saeki et al., 2020). Our results confirmed the anti-QS ability of EGCG against *P. aeruginosa in vivo*. EGCG significantly inhibited the expression of QS-relevant genes, including *lasI*, *lasR*, *rhlI*, *rhlR*, *pqsA*, *pqsR*, *phzA*, *phzH*, *phzM*, *phzS*, *lasA*, *lasB*, *rhlA*, and *rhlC*. Moreover, EGCG had a better capacity to inhibit virulence than the positive CIP group. According to the gene expression results, EGCG displayed an excellent ability to inhibit proinflammatory cytokines. At 80 mg/kg, EGCG significantly increased anti-inflammatory cytokines IL-10 and IL-4, which was not observed in the CIP group. We found that the expression of *lasR*/*lasI* and *rhlR*/*rhlI* systems, the leading virulence factor during *P. aeruginosa* infection, was significantly inhibited at 80 mg/kg. Pyocyanin, a green color product of *P. aeruginosa*, can interfere with host oxidative stress responses by increasing intracellular levels of reactive oxygen species (Lau et al., 2004). Our results show that the inhibition ability of EGCG on the expression of QS-regulated virulence factors was better than that of CIP, especially in the QS systems *lasR*/*lasI*, *rhlR*/*rhlI* and *pqsR*/*pqsA*. Thus, these results suggested that EGCG not only alleviates inflammation but also reduces the damage of *P. aeruginosa* infection by targeting QS system-regulated virulence, thereby resulting in a higher protective effect than CIP. EGCG is a great potential QS inhibitor against infection that not only reduces the release of virulence factors *in vitro* but also effectively inhibits the expression of virulence factors *in vivo*.

## Conclusion

In conclusion, our results provide proof that EGCG alone can ameliorate acute *P. aeruginosa* infection in the lungs by QS and inflammation inhibition. The *in vivo* results indicate that the QS inhibitor, EGCG,s may serve as a potential approach in treating bacterial infections. EGCG, a rich ingredient of tea, is indispensable to human life and may play an important role in preventing bacterial infections.

## Data Availability Statement

The raw data supporting the conclusions of this article will be made available by the authors, without undue reservation.

## Ethics Statement

The animal study was reviewed and approved by Animal Ethical Committee of Sichuan Agricultural University.

## Author Contributions

HT and SH contributed to conceptualization, methodology, validation, and investigation. LZ and FS contributed to formal analysis and investigation. YL, QW, HG, CL, JL, and ZZ contributed to writing—original draft and resources. GY contributed to writing—review and editing, supervision, resources, and funding acquisition. All authors contributed to the article and approved the submitted version.

## Funding

This work was supported by International Science and Technology Cooperation Program of Sichuan: 2020YFH0143; Traditional Chinese Veterinary Medicine Laboratory of the National Ethnic Affairs Commission [2020] 03.

## Conflict of Interest

The authors declare that the research was conducted in the absence of any commercial or financial relationships that could be construed as a potential conflict of interest.

## Publisher’s Note

All claims expressed in this article are solely those of the authors and do not necessarily represent those of their affiliated organizations, or those of the publisher, the editors and the reviewers. Any product that may be evaluated in this article, or claim that may be made by its manufacturer, is not guaranteed or endorsed by the publisher.

## References

[ref1] Aloush V. Navon-Venezia S. Seigman-Igra Y. Cabili S. Carmeli Y. (2006). Multidrug-resistant Pseudomonas aeruginosa: risk factors and clinical impact. Antimicrob. Agents Chemother. 50, 43–48. doi: 10.1128/AAC.50.1.43-48.2006, PMID: 16377665 PMC1346794

[ref2] Azambuja J. H. Mancuso R. I. Della Via F. I. Torello C. O. Saad S. T. O. (2022). Protective effect of green tea and epigallocatechin-3-gallate in a LPS-induced systemic inflammation model. J. Nutr. Biochem. 101:108920. doi: 10.1016/j.jnutbio.2021.108920, PMID: 34875388

[ref3] Balasubramanian D. Schneper L. Kumari H. Mathee K. (2013). A dynamic and intricate regulatory network determines Pseudomonas aeruginosa virulence. Nucleic Acids Res. 41, 1–20. doi: 10.1093/nar/gks1039, PMID: 23143271 PMC3592444

[ref4] Breidenstein E. B. de la Fuente-Núñez C. Hancock R. E. (2011). Pseudomonas aeruginosa: all roads lead to resistance. Trends Microbiol. 19, 419–426. doi: 10.1016/j.tim.2011.04.005, PMID: 21664819

[ref5] Chatterjee M. Anju C. Biswas L. Kumar V. A. Mohan C. G. Biswas R. (2016). Antibiotic resistance in Pseudomonas aeruginosa and alternative therapeutic options. Int. J. Med. Microbiol. 306, 48–58. doi: 10.1016/j.ijmm.2015.11.004 26687205

[ref6] Clatworthy A. E. Pierson E. Hung D. T. (2007). Targeting virulence: a new paradigm for antimicrobial therapy. Nat. Chem. Biol. 3, 541–548. doi: 10.1038/nchembio.2007.24, PMID: 17710100

[ref7] Costerton J. W. Stewart P. S. Greenberg E. P. (1999). Bacterial biofilms: a common cause of persistent infections. Science 284, 1318–1322. doi: 10.1126/science.284.5418.1318 10334980

[ref8] Crousilles A. Maunders E. Bartlett S. Fan C. Ukor E.-F. Abdelhamid Y.. (2015). Which microbial factors really are important in Pseudomonas aeruginosa infections? Future Microbiol. 10, 1825–1836. doi: 10.2217/fmb.15.100, PMID: 26515254

[ref9] Dai R.-X. Kong Q.-H. Mao B. Xu W. Tao R.-J. Wang X.-R.. (2018). The mortality risk factor of community acquired pneumonia patients with chronic obstructive pulmonary disease: a retrospective cohort study. BMC Pulm. Med. 18, 12–10. doi: 10.1186/s12890-018-0587-7, PMID: 29357862 PMC5778745

[ref10] Driscoll J. A. Brody S. L. Kollef M. H. (2007). The epidemiology, pathogenesis and treatment of Pseudomonas aeruginosa infections. Drugs 67, 351–368. doi: 10.2165/00003495-200767030-00003 17335295

[ref11] Gellatly S. L. Hancock R. E. (2013). Pseudomonas aeruginosa: new insights into pathogenesis and host defenses. Path. Dis. 67, 159–173. doi: 10.1111/2049-632X.12033, PMID: 23620179

[ref12] Hancock R. E. Speert D. P. (2000). Antibiotic resistance in Pseudomonas aeruginosa: mechanisms and impact on treatment. Drug Resist. Updat. 3, 247–255. doi: 10.1054/drup.2000.0152 11498392

[ref13] Hao S. Yang D. Zhao L. Shi F. Ye G. Fu H.. (2021). EGCG-mediated potential inhibition of biofilm development and quorum sensing in Pseudomonas aeruginosa. Int. J. Mol. Sci. 22:4946. doi: 10.3390/ijms22094946, PMID: 34066609 PMC8125375

[ref14] Hauser A. R. (2009). The type III secretion system of Pseudomonas aeruginosa: infection by injection. Nat. Rev. Microbiol. 7, 654–665. doi: 10.1038/nrmicro2199, PMID: 19680249 PMC2766515

[ref15] Jain S. Self W. H. Wunderink R. G. Fakhran S. Balk R. Bramley A. M.. (2015). Community-acquired pneumonia requiring hospitalization among US adults. N. Engl. J. Med. 373, 415–427. doi: 10.1056/NEJMoa1500245, PMID: 26172429 PMC4728150

[ref16] Jigisha A. Nishant R. Navin K. Pankaj G. (2012). Green tea: a magical herb with miraculous outcomes. Int. Res. J. Pharm 3, 139–148.

[ref17] Kipnis E. Sawa T. Wiener-Kronish J. (2006). Targeting mechanisms of Pseudomonas aeruginosa pathogenesis. Med. et Mal. Infect. 36, 78–91. doi: 10.1016/j.medmal.2005.10.007 16427231

[ref18] Kizny Gordon A. E. Mathers A. J. Cheong E. Y. Gottlieb T. Kotay S. Walker A. S.. (2017). The hospital water environment as a reservoir for carbapenem-resistant organisms causing hospital-acquired infections—a systematic review of the literature. Clin. Infect. Dis. 64, 1435–1444. doi: 10.1093/cid/cix132, PMID: 28200000

[ref19] Lau G. W. Hassett D. J. Ran H. Kong F. (2004). The role of pyocyanin in Pseudomonas aeruginosa infection. Trends Mol. Med. 10, 599–606. doi: 10.1016/j.molmed.2004.10.002 15567330

[ref20] Lee I.-T. Lin C.-C. Lee C.-Y. Hsieh P.-W. Yang C.-M. (2013). Protective effects of (−)-epigallocatechin-3-gallate against TNF-α-induced lung inflammation via ROS-dependent ICAM-1 inhibition. J. Nutr. Biochem. 24, 124–136. doi: 10.1016/j.jnutbio.2012.03.009, PMID: 22819551

[ref21] Lee J. Zhang L. (2015). The hierarchy quorum sensing network in Pseudomonas aeruginosa. Protein Cell 6, 26–41. doi: 10.1007/s13238-014-0100-x, PMID: 25249263 PMC4286720

[ref22] Ling J.-X. Wei F. Li N. Li J.-L. Chen L.-J. Liu Y.-Y.. (2012). Amelioration of influenza virus-induced reactive oxygen species formation by epigallocatechin gallate derived from green tea. Acta Pharmacol. Sin. 33, 1533–1541. doi: 10.1038/aps.2012.80 22941291 PMC4001843

[ref23] Liu W. Lu H. Chu X. Lou T. Zhang N. Zhang B.. (2020). Tea polyphenols inhibits biofilm formation, attenuates the quorum sensing-controlled virulence and enhances resistance to Klebsiella pneumoniae infection in Caenorhabditis elegans model. Microb. Pathog. 147:104266. doi: 10.1016/j.micpath.2020.104266, PMID: 32442664

[ref24] Livermore D. M. (2002). Multiple mechanisms of antimicrobial resistance in Pseudomonas aeruginosa: our worst nightmare? Clin. Infect. Dis. 34, 634–640. doi: 10.1086/338782, PMID: 11823954

[ref25] Melsen W. G. Rovers M. M. Groenwold R. H. Bergmans D. C. Camus C. Bauer T. T.. (2013). Attributable mortality of ventilator-associated pneumonia: a meta-analysis of individual patient data from randomised prevention studies. Lancet Infect. Dis. 13, 665–671. doi: 10.1016/S1473-3099(13)70081-1, PMID: 23622939

[ref26] Moore N. M. Flaws M. L. (2011). Antimicrobial resistance mechanisms in Pseudomonas aeruginosa. Clin. Lab. Sci. 24, 47–51. doi: 10.29074/ascls.24.1.47 21404965

[ref27] Nadal Jimenez P. Koch G. Thompson J. A. Xavier K. B. Cool R. H. Quax W. J. (2012). The multiple signaling systems regulating virulence in Pseudomonas aeruginosa. Microbiol. Mol. Biol. Rev. 76, 46–65. doi: 10.1128/MMBR.05007-11, PMID: 22390972 PMC3294424

[ref28] O’Brien K. L. Baggett H. C. Brooks W. A. Feikin D. R. Hammitt L. L. Higdon M. M.. (2019). Causes of severe pneumonia requiring hospital admission in children without HIV infection from Africa and Asia: the PERCH multi-country case-control study. Lancet 394, 757–779. doi: 10.1016/S0140-6736(19)30721-4, PMID: 31257127 PMC6727070

[ref29] O’Neill J. (2016). Tackling drug-resistant infections globally: final report and recommendations.

[ref30] Pylaeva E. Bordbari S. Spyra I. Decker A. S. Häussler S. Vybornov V.. (2019). Detrimental effect of type I IFNs During acute lung infection With Pseudomonas aeruginosa is mediated Through the stimulation of neutrophil NETosis. Front. Immun. 10:2190, 2190. doi: 10.3389/fimmu.2019.02190, PMID: 31572395 PMC6749149

[ref31] Quinton L. J. Mizgerd J. P. (2015). Dynamics of lung defense in pneumonia: resistance, resilience, and remodeling. Annu. Rev. Physiol. 77, 407–430. doi: 10.1146/annurev-physiol-021014-071937, PMID: 25148693 PMC4366440

[ref32] Ravi Kumar S. Paudel S. Ghimire L. Bergeron S. Cai S. Zemans R. L.. (2018). Emerging roles of inflammasomes in acute pneumonia. Am. J. Respir. Crit. Care Med. 197, 160–171. doi: 10.1164/rccm.201707-1391PP, PMID: 28930487 PMC5768907

[ref33] Sadikot R. T. Blackwell T. S. Christman J. W. Prince A. S. (2005). Pathogen–host interactions in Pseudomonas aeruginosa pneumonia. Am. J. Respir. Crit. Care Med. 171, 1209–1223. doi: 10.1164/rccm.200408-1044SO, PMID: 15695491 PMC2718459

[ref34] Saeki E. K. Kobayashi R. K. T. Nakazato G. (2020). Quorum sensing system: target to control the spread of bacterial infections. Microb. Pathog. 142:104068. doi: 10.1016/j.micpath.2020.104068, PMID: 32061914

[ref35] Sauer K. Camper A. K. Ehrlich G. D. Costerton J. W. Davies D. G. (2002). Pseudomonas aeruginosa displays Multiple phenotypes during development as a biofilm. SAM J. 184, 1140–1154. doi: 10.1128/jb.184.4.1140-1154.2002, PMID: 11807075 PMC134825

[ref36] Sen-Kilic E. Blackwood C. B. Boehm D. T. Witt W. T. Malkowski A. C. Bevere J. R.. (2019). Intranasal peptide-based FpvA-KLH conjugate vaccine protects mice from Pseudomonas aeruginosa acute murine pneumonia. Front. Immunol. 10:2497. doi: 10.3389/fimmu.2019.02497, PMID: 31708925 PMC6819369

[ref37] Shapouri-Moghaddam A. Mohammadian S. Vazini H. Taghadosi M. Esmaeili S. A. Mardani F.. (2018). Macrophage plasticity, polarization, and function in health and disease. J. Cell. Physiol. 233, 6425–6440. doi: 10.1002/jcp.26429, PMID: 29319160 PMC13482560

[ref38] Sharma A. Vaghasiya K. Ray E. Gupta P. Gupta U. D. Singh A. K.. (2020). Targeted pulmonary delivery of the green tea polyphenol Epigallocatechin Gallate controls the growth of mycobacterium tuberculosis by enhancing the autophagy and suppressing bacterial burden. ACS Biomater Sci. Eng. 6, 4126–4140. doi: 10.1021/acsbiomaterials.0c00823, PMID: 33463343

[ref39] Skariyachan S. Sridhar V. S. Packirisamy S. Kumargowda S. T. Challapilli S. B. (2018). Recent perspectives on the molecular basis of biofilm formation by Pseudomonas aeruginosa and approaches for treatment and biofilm dispersal. Folia Microbiol. 63, 413–432. doi: 10.1007/s12223-018-0585-4, PMID: 29352409

[ref40] Sriram N. Kalayarasan S. Sudhandiran G. (2009). Epigallocatechin-3-gallate exhibits anti-fibrotic effect by attenuating bleomycin-induced glycoconjugates, lysosomal hydrolases and ultrastructural changes in rat model pulmonary fibrosis. Chem. Biol. Interact. 180, 271–280. doi: 10.1016/j.cbi.2009.02.017, PMID: 19497426

[ref41] Steinmann J. Buer J. Pietschmann T. Steinmann E. (2013). Anti-infective properties of epigallocatechin-3-gallate (EGCG), a component of green tea. Br. J. Pharmacol. 168, 1059–1073. doi: 10.1111/bph.12009, PMID: 23072320 PMC3594666

[ref42] Traber K. E. Dimbo E. L. Symer E. M. Korkmaz F. T. Jones M. R. Mizgerd J. P.. (2019). Roles of interleukin-11 during acute bacterial pneumonia. PLoS One 14:e0221029. doi: 10.1371/journal.pone.0221029, PMID: 31415618 PMC6695241

[ref43] Wang J. Fan S. M. Zhang J. (2019). Epigallocatechin-3-gallate ameliorates lipopolysaccharide-induced acute lung injury by suppression of TLR4/NF-κB signaling activation. Braz. J. Med. Biol. Res. 52:e8092. doi: 10.1590/1414-431x20198092, PMID: 31241712 PMC6596362

[ref44] Yang D. Hao S. Zhao L. Shi F. Ye G. Zou Y.. (2021). Paeonol attenuates quorum-sensing regulated virulence and biofilm formation in Pseudomonas aeruginosa. Front. microbiol. 12:692474. doi: 10.3389/fmicb.2021.692474, PMID: 34421847 PMC8371487

[ref45] Zhao Z. Meiyan F. Juan W. Zheng X. Teng C. Xinya X.. (2021). Research Progress of Epigallocatechin-3-gallate (EGCG) on anti-pathogenic microbes and immune regulation. Food Funct. 12, 9607–9619. doi: 10.1039/D1FO01352A 34549212

